# Recruitment of Immune Cells into Inflamed Tissues: Consequences for Endothelial Barrier Integrity and Tissue Functionality

**DOI:** 10.1155/2016/1561368

**Published:** 2016-02-17

**Authors:** Michael Schnoor, Pilar Alcaide, Mathieu-Benoit Voisin, Jaap D. van Buul

**Affiliations:** ^1^Department of Molecular Biomedicine, Center for Investigation and Advanced Studies of the National Polytechnic Institute (Cinvestav), 07360 Mexico City, DF, Mexico; ^2^Molecular Cardiology Research Institute, Tufts Medical Center and Tufts University School of Medicine, Boston, MA 02111, USA; ^3^Centre for Microvascular Research, William Harvey Research Institute, Barts & The London SMD, Queen Mary University of London, London EC1M 6BQ, UK; ^4^Department of Molecular Cell Biology, Sanquin Research and Landsteiner Laboratory, Academic Medical Center, University of Amsterdam, 1066 CX Amsterdam, Netherlands

Immune cell trafficking is critical for survival. In order to fulfil their functions in surveillance and immune responses, leukocytes need to exit the blood stream to reach sites of infection or injury in peripheral tissues [1]. Once transmigrated, leukocytes trigger pathogen clearance, resolution of inflammation, and wound healing. On the other hand, excessive extravasation of leukocytes, as seen in pathological inflammatory conditions including sepsis, autoimmune, and cardiovascular diseases, can provoke tissue damage due to excessive release of proinflammatory cytokines, chemokines, and reactive oxygen species. These substances cause endothelial overactivation triggering leukocyte transmigration and endothelial dysfunction [2]. The complex nature of physiological and pathological leukocyte extravasation makes it tricky to define a threshold where beneficial transmigration ends and where detrimental excessive extravasation starts. Thus, it is of utmost importance to better understand the mechanisms that control leukocyte-endothelial interactions during the extravasation cascade and leukocyte behaviour within peripheral tissues that ensure appropriate immune responses. The purpose of this special issue was to publish original research and review articles addressing recent conceptual advances in the field of inflammatory immune cell recruitment, endothelial dysfunction, and resolution of inflammation.

The editors contributed a review article in which they highlight certain mechanisms that different subsets exploit to achieve the goal of transmigration. This is actually very important when it comes to the treatment of immune diseases caused by a certain leukocyte subset. In this case, it is desired to only interfere with the transmigration of this certain subset without affecting others to avoid complete immune suppression that would cause severe side effects. Such mechanisms include tyrosine phosphorylation of endothelial adhesion receptors that are summarized in the review by Dr. A. P. Adam. He highlights signalling pathways downstream of proinflammatory cytokines and adhering leukocytes that induce tyrosine phosphorylation of junctional proteins such as VE-cadherin. He discusses the consequences of these phosphorylations that may or may not lead to junction disassembly and excessive leukocyte transmigration. J. Kryczka and J. Boncela nicely review the multidimensional impact of leukocytes in skin fibrosis emphasizing current knowledge about recruitment of different leukocyte types in wounded tissues and the mechanisms triggering wound enclosure. A fine balance between scar formation and massive fibrosis is required to avoid excessive fibrosis while promoting wound healing. M. C. Souza and colleagues present a comprehensive review on endothelial dysfunction in cerebral malaria. The authors summarize known leukocyte interactions with the brain and lung endothelium and highlight the challenges of using experimental malaria data in animals to interpret and understand human severe malaria. Moreover, the J. Millán group contributed a comprehensive review on how leukocytes, once extravasated, interact with polarized epithelial cells in inflamed tissues. They emphasize that polarized epithelial cells not only secrete chemokines asymmetrically, but also polarize adhesion receptors that may guide leukocytes across epithelial monolayers. In the review of J. Cedervall and colleagues the latest findings on the alterations of vascular functions by tumor cells and tumor-associated leukocytes are summarized. They also discuss how circulating tumor-derived factors drive systemic inflammation in the blood vasculature of distant organs.

In a clinical study, A. Lukasz and colleagues elegantly showed that plasma angiopoietin-2 levels may have predictive value for a complicated clinical course of hemolytic uremic syndrome. Using endothelial cells in vitro, the authors showed that serum from these patients reduced barrier stability. S. Denk and coworkers showed the diagnostic implications of the presence of JAM-1 in the circulation for the detection of experimental and clinical polytrauma. C. Kirchhoff and colleagues developed a method that may potentially help identifying traumatic brain infarction by showing that monocytes accumulate in the circulation shortly after onset of the infarct. In another research article, E. Gallego-Colon and colleagues demonstrate that the number of infiltrating proinflammatory monocytes decreased in the heart after myocardial infarction whereas the number of anti-inflammatory macrophages increased in mice overexpressing insulin-like growth factor-1Ea (IGF-1Ea) leading to reduced scar formation and increased cardiac functional recovery. These data highlight the importance of the balance between pro- and anti-inflammatory leukocytes for tissue repair after myocardial infarction, which is in part controlled by IGF-1Ea. S. A. Dorosz and colleagues provide new evidence that IL-27 promotes proinflammatory signals in endothelial cells that are downregulated by the damage-associated molecular pattern molecule calprotectin through mechanisms involving STAT-1. These findings highlight calprotectin as a modulator of inflammation and early vascular dysfunction.

We hope that this special issue will be beneficial for clinicians and researchers not only in the field of leukocyte transmigration but also for those working on acute and chronic inflammatory diseases. The studies published in this special issue provide new insights into physiologically relevant mechanisms of inflammation and inflammatory diseases that will hopefully stimulate the development of novel research ideas and therapeutic strategies.
